# Understanding the Brassinosteroid-Dependent Environmental Adaption in Brassicaceae Plants

**DOI:** 10.3390/plants14101554

**Published:** 2025-05-21

**Authors:** Zhenni Lu, Changrui Ma, Yuzhen Xie, Yuqing Zeng, Jiashi Peng, Dinggang Zhou, Jinfeng Wu

**Affiliations:** 1 School of Life and Health Sciences, Hunan University of Science and Technology, Xiangtan 411201, China; lJenny0725@163.com (Z.L.); changruima@foxmail.com (C.M.); xyz11238011@163.com (Y.X.); 18670907635@163.com (Y.Z.); jspeng@hnust.edu.cn (J.P.); dgzhoucn@hnust.edu.cn (D.Z.); 2 Hunan Key Laboratory of Economic Crops Genetic Improvement and Integrated Utilization, Hunan University of Science and Technology, Xiangtan 411201, China; 3 Yuelushan Laboratory, Changsha 410125, China

**Keywords:** Brassinosteroids, stress, crosstalk, adaption, Brassicaceae crop

## Abstract

Plant adaptation to various stresses depends on transmitting the external stress signals into internal signals. Brassinosteroids (BRs) play pivotal roles in connecting the external and internal signals in Brassicaceae plants, particularly under abiotic stresses such as drought, cold, heat and salinity. They modulate plant growth and stress responses through receptor kinase-mediated signaling pathways, which integrate with redox homeostasis, antioxidant systems and crosstalk with other phytohormones, including auxin, abscisic acid, ethylene, cytokinins, gibberellines, jasmonates and salicylic acid. BR-dependent pathways are critical for balancing stress resilience and productivity in Brassicaceae plants. In this review, we introduce BR metabolism, signaling transduction and discuss their functions in regulating growth and development processes under adverse environment in Brassicaceae plants. We also emphasize recent advances in the crosstalk among BR and other phytohormones in stresses response. Understanding the mechanisms of BR-dependent pathways offers new approaches for enhancing the adaptation under adverse conditions in Brassicaceae crops.

## 1. Introduction

The plant steroid hormone Brassinosteroid (BR) is the sixth major plant hormone discovered after auxin, gibberellin, cytokinin, abscisic acid (ABA) and ethylene [1]. In 1979, scientists collected 227 kg of rapeseed pollen, from which they isolated and purified 4 mg of brassinolide, which is the first determination of the molecular structure of brassinolide, and officially named it as brassinolide (BL), also known as brassinosteroid [2]. Approximately seventy natural brassinosteroid compounds have been isolated and identified from plants [3]. Among these, the most potent are BL and 24-epibrassinolide (24-EBL). Natural BRs are widely distributed in various plants, such as rapeseed, wheat and rice. They are mainly found in rapidly growing parts, including pollen, seeds and shoot tips [4]. Meanwhile, there are also some artificially synthesized brassinosteroid analogs, such as 24-BL, and the synthesis methods and biological activities of 24-EBL have been extensively studied [5]. BRs play an important role in plant growth and development, especially in promoting cell growth, enhancing stress resistance and regulating growth and development [6,7]. They play a significant regulatory role in a wide range of physiological processes in plants, from seed development to the regulation of flowering and senescence [8]. They can control cell differentiation and the formation of tissue patterns. BRs also play a regulatory role in controlling cell cycle progression and differentiation in the root meristem of arabidopsis [9]. BRs control the size of the meristem by promoting the cell cycle progression in roots in arabidopsis [10]. It was found that *BnDF4* encodes brassinosteroid-insensitive 2 (BIN2) in rapeseed, which regulates plant height by blocking the elongation of basal internode cells [11]. Additionally, BRs play important roles in flower development and fruit ripening. Studies have shown that the BR signaling pathway regulates flowering time by recruiting histone demethylase enzymes [12]. Moreover, it was found that BRs can enhance the efficiency of photosynthesis in plants and promote the development of their chloroplasts [13]. Some studies have also shown that BRs function to stabilize squamosa promoter binding protein-like 9 (SPL9) and target of early activation tagged 1 (EAT1) simultaneously to regulate the vegetative phase change in plants [14].

BRs play critical roles not only in adapting to the environment but also in interacting with other hormones in Brassicaceae plants. Recent studies have shown that exogenous BR treatment during the early seed germination stage of rapeseed can up-regulate the expression of auxin-related genes, thereby promoting the establishment of rapeseed seedlings [15]. Some publications have provided detailed and comprehensive information on the biosynthesis of BRs, including the most active form, BL, and its production process [16]. Another review article introduce the roles of BR in plant growth, development and stress responses [17]. However, the understanding of BR’s functions in Brassicaceae crops, especially in terms of environmental adaptability, remains less well known. This situation hinders its application in the production of Brassicaceae. We provide an overview of the biosynthesis, metabolism and signaling pathways of BR and its impacts on the growth and development of Brassicaceae plants. Then we explore the complex interaction networks formed between BRs and other phytohormones in signaling pathways and their regulatory effects on the growth and development of Brassicaceae plants. Furthermore, we delve into the functional roles of BR in the environmental adaptability of Brassicaceae crops (*Raphanus sativus* L., *Brassica rapa* L., *Brassica oleracea* L., *Brassica napus* L., and *Brassica juncea* L.), such as how BR alleviates the adverse effects of drought, temperature and other stresses in plants. This review provides important insights into the application of BRs in Brassicaceae crops, especially in terms of environmental adaptability.

## 2. The Synthesis and Metabolism of BRs

### 2.1. The Synthesis of BRs

**Figure 1:**
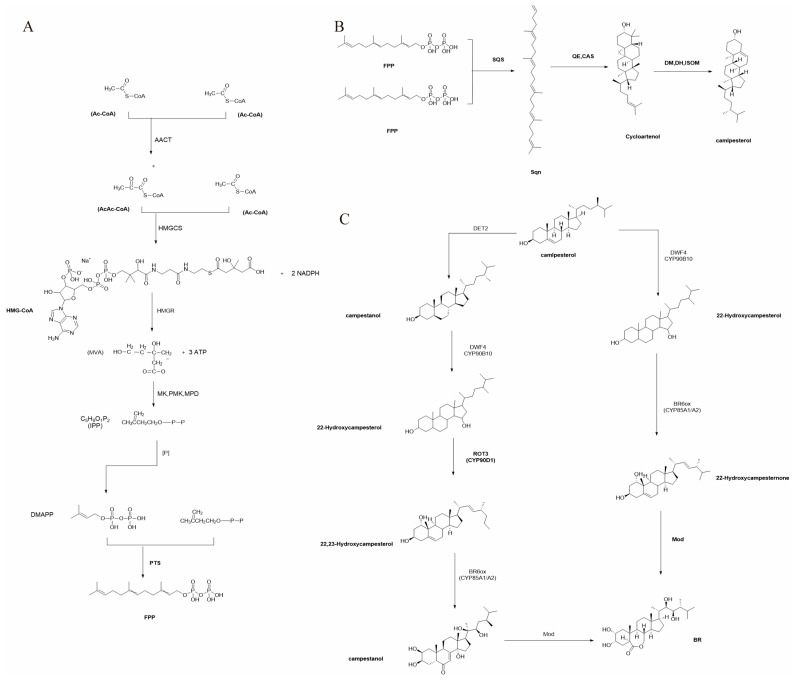
The biosynthesis of BRs in plants. (**A**) the generation of brassinosteroid precursors, specifically FPP, which includes the following steps: (1) Two molecules of A-CoA are condensed to form AA-CoA, a process catalyzed by acetoacetyl-CoA thiolase (AACT). (2) AA-CoA then combines with another molecule of A-CoA to produce HMG-CoA, catalyzed by HMG-CoA synthase (HMGCS). (3) HMG-CoA is subsequently reduced to mevalonate (MVA) by HMG-CoA reductase (HMGR), utilizing two molecules of NADPH. (4) MVA undergoes three consecutive phosphorylation reactions, catalyzed by mevalonate kinase (MK), phosphomevalonate kinase (PMK), and mevalonate diphosphate decarboxylase (MPD), to IPP. (5) IPP is partially converted to dimethylallyl diphosphate (DMAPP) by IPI. Finally, IPP and DMAPP are condensed by prenyltransferases (PTS) to form the brassinosteroid precursor FPP; (**B**) The generation of Campesterol, which involves: (1) The condensation of two molecules of FPP to form squalene (Sqn), catalyzed by squalene synthase (SQS). (2) Sqn is then oxidized to 2,3-oxidosqualene by squaleneepoxidase (SQE). (3) 2,3-Oxidosqualene is converted to cycloartenol by cycloartenol synthase (CAS). (4) Cycloartenol (Cycloart) undergoes a series of demethylation, dehydrogenation, and isomerization reactions to ultimately form campesterol (Cmpt); (**C**) The generation of BRs, which can occur through two pathways: (1) The CN-dependent pathway, which includes: (a) Cmpt is reduced to CN by 5α-reductase (DET2). (b) CN is hydroxylated at the C-22 position by C-22 hydroxylase (DWF4/CYP90B1) to form 22-hydroxycampestanol (22-OH-Cmpt). (c) 22-OH-Cmpt is further hydroxylated at the C-23 position by C-23 hydroxylase (ROT3/CYP90D1) to form 22,23-dihydroxycampestanol (22,23-OH-Cmpt). (d) 22,23-OH-Cmpt is oxidized at the C-6 position by C-6 oxidase (BR6ox/CYP85A1/A2) to form CS. (e) CS is further modified to form the bioactive R. (2) The non-CN-dependent pathway, which includes: (a) CN is directly hydroxylated at the C-22 position by DWF4/CYP90B1 to form 22-OH-Cmpt. (b) 22-OH-Cmpt is oxidized at the BR6ox/CYP85A1/A2 to form 22-OHCmpt. (c) 22-OHCmpt undergoes a series of modifications to ultimately form the bioactive BR.

The biosynthesis of BRs in plants involves three main stages. First, the generation of the precursor Farnesyl Pyrophosphate (FPP) occurs through a series of reactions starting with the condensation of acetyl-CoA (A-CoA) to form aceto-acetyl-CoA (AA-CoA) and then 3-hydroxy-3-methylglutaryl-CoA (HMG-CoA), which is reduced to mevalonate and converted to isoprenoid diphosphate (IPP). IPP is converted to dimethylallyl diphosphate (DMAPP) and then condensed to form FPP [18]. Then campesterol is converted to BRs through two pathways: the campestanol (CN)-dependent pathway, which includes multiple hydroxylation and oxidation steps to form bioactive BRs, and the non-CN-dependent pathway, which directly hydroxylates campesterol and undergoes further modifications to produce active BRs [19]. The synthesis of campesterol begins with the condensation of three A-CoA molecules to form mevalonic acid (MVA). MVA is then converted into isoprenoid pyrophosphates, IPP and FPP. Subsequently, two molecules of FPP combine to form squalene (C30). Finally, squalene undergoes cyclization to form cycloartenol, which is then converted to campesterol through demethylation, dehydrogenation, and isomerization reactions [20]. In the CN-dependent pathway, campesterol is first reduced to CN, which is then oxidized at the C-6 position to form 6-oxocampestanol. Subsequently, 6-oxocampestanol is further converted to teasterone (TE), typhasterol (TY), and ultimately to the final precursor, castasterone (CS). The non-campestanol-dependent pathway. Campesterol is directly hydroxylated at the C-22 position by DWF4 without being converted to campestanol, and then enters the late C-6 oxidation pathway. This pathway is dominant in plants such as Arabidopsis thaliana, as DWF4 has a higher affinity for campesterol as its substrate. Under light conditions, plants tend to favor the late C-6 oxidation pathway [21] (Figure 1).

### 2.2. The Degradation of BRs

In the CN-dependent pathway, CR is first reduced to CN, which is then oxidized at the C-6 position to form 6-oxocampestanol. This intermediate is further converted to TE, TY, and ultimately to the precursor CS [22]. In the non-campestanol-dependent pathway, campesterol is directly hydroxylated at the C-22 position by dwarf4 (DWF4) without being converted to campestanol and then enters the late C-6 oxidation pathway [23]. This pathway is dominant in plants such as Arabidopsis thaliana because DWF4 has a higher affinity for campesterol as its substrate. The biosynthesis of BRs also involves the transformation of other intermediates, such as the oxidation of cholestanol to 6-oxocholestanol, and the synthesis from 28-norcastasterone to 28-norbrassinolide [24]. Additionally, the C-22 oxidation pathway is also an important branch in the biosynthesis of BRs [25].

The biosynthesis of BRs in plants primarily involves three stages. First is the generation of brassinosteroid precursors, specifically FPP, which includes the following steps. (1) Two molecules of A-CoA are condensed to form AA-CoA, a process catalyzed by aceto-acetyl-CoA thiolase (AACT). (2) AA-CoA then combines with another molecule of A-CoA to produce HMG-CoA, catalyzed by HMG-CoA synthase (HMGCS). (3) HMG-CoA is subsequently reduced to mevalonate (MVA) by HMG-CoA reductase (HMGR), utilizing two molecules of NADPH. (4) MVA undergoes three consecutive phosphorylation reactions, catalyzed by mevalonate kinase (MK), phosphomevalonate kinase (PMK), and mevalonate diphosphate decarboxylase (MPD), to IPP. (5) IPP is partially converted to dimethylallyl diphosphate (DMAPP) by IPI. Finally, IPP and DMAPP are condensed by prenyl-transferases (PTS) to form the brassinosteroid precursor FPP. The second stage is the generation of Campesterol, which involves the following. (1) The condensation of two molecules of FPP to form squalene (Sqn), catalyzed by squalene synthase (SQS). (2) Sqn is then oxidized to 2,3-oxidosqualene by squalene-epoxidase (SQE). (3) 2,3-Oxidosqualene is converted to cycloartenol by cycloartenol synthase (CAS). (4) Cycloartenol (Cycloart) undergoes a series of demethylation, dehydrogenation, and isomerization reactions to ultimately form campesterol (Cmpt). The third stage is the generation of BRs, which can occur through two pathways. (1) The CN-dependent pathway, which includes: (a) Cmpt is reduced to CN by 5α-reductase (DET2). (b) CN is hydroxylated at the C-22 position by C-22 hydroxylase (DWF4/CYP90B1) to form 22-hydroxycampestanol (22-OH-Cmpt). (c) 22-OH-Cmpt is further hydroxylated at the C-23 position by C-23 hydroxylase (ROT3/CYP90D1) to form 22,23-dihydroxycampestanol (22,23-OH-Cmpt). (d) 22,23-OH-Cmpt is oxidized at the C-6 position by C-6 oxidase (BR6ox/CYP85A1/A2) to form CS. (e) CS is further modified to form the bioactive R. (2) The non-CN-dependent pathway involves the following. (a) CN is directly hydroxylated at the C-22 position by DWF4/CYP90B1 to form 22-OH-Cmpt. (b) 22-OH-Cmpt is oxidized at the BR6ox/CYP85A1/A2 to form 22-OHCmpt. (c) 22-OHCmpt undergoes a series of modifications to ultimately form the bioactive BR.

## 3. BR Signal Transduction

### 3.1. BR Receptor Activation and Recognition

**Figure 2:**
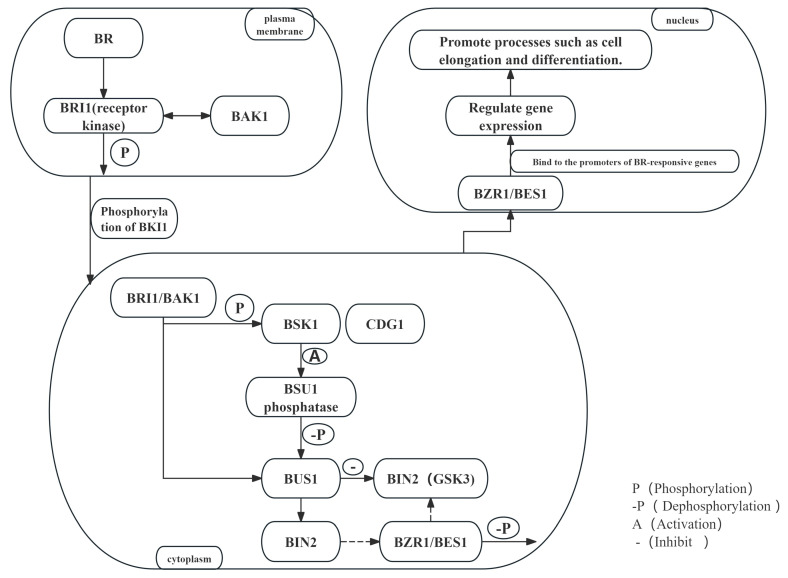
BR signaling pathway.

BRs bind to the receptor kinase brassinosteroid insensitive 1 (BRI1) and its co-receptor bri1-associated kinase 1 (BAK1) on the cell membrane, triggering the formation of an active BRI1-BAK1 complex [26]. The interaction and mutual phosphorylation of BRI1 and BAK1 enhance signal transduction and initiate downstream cascades [27]. In the absence of BRs, BRI1 interacts with bri1 kinase inhibitor 1 (BKI1) and bri1 inhibitor-related (BIR) proteins, which inhibit the formation of the BRI1-BAK1 complex [28]. When BRs are secreted into the extracellular matrix via the atp-binding cassette subfamily b 1/19 (ABCB1/19) transporter on the cell membrane, they are perceived by the BRI1-BAK1 complex, leading to the dissociation of BKI1 from BRI1 [29,30] (Figure 2).

### 3.2. The Downstream Signaling Cascade of BRs

Activated BRI1 phosphorylates and activates downstream receptor-like cytoplasmic kinases include br signaling kinase 1 (BSK1) and constitutive differential growth 1 (CDG1) [31]. These kinases further phosphorylate bri1 suppressor 1 (BSU1), which dephosphorylates and inactivates BIN2, a GSK3-like protein kinase [32]. The inactivation of BIN2 leads to the accumulation of non-phosphorylated forms of transcription factors, such as bri1-ems-suppressor 1 (BES1) and brassinazole resistant 1 (BZR1), in the nucleus (Figure 2) [33].

### 3.3. BR-Mediated Transcriptional Regulation and Gene Expression

After non-phosphorylated BES1 and BZR1 enter the nucleus, they regulate plant growth and development by activating the transcription of downstream BR-responsive genes [34]. Additionally, factors such as 14-3-3 proteins and brz-sensitive-short hypocotyl 1 (BSS1) regulate the phosphorylation status and localization of BES1/BZR1 in the cytoplasm [35]. In addition, several studies have shown that proteins such as twisted dwarf1 (TWD1), plant u-box e3 ubiquitin ligases 12/13 (PUB12/130), ubiquitin-specific protease 12/13 (UBP12/13), kink suppressed in ber1 (KIB1) and the receptor for activated c kinase 1 (RACK1) play important roles in BR signaling transduction. For instance, there are studies indicating that TWD1 plays a crucial role in BR-induced interaction between BRI1 and its co-receptor BAK1, as well as in the BR-induced phosphorylation of these two proteins [36]. PUB12 and PUB13, acting as E3 ubiquitin ligases, are capable of mediating the ubiquitination and degradation of BRI1, thereby regulating the intensity of BR signaling [37]. UBP12 and UBP13, acting as deubiquitinases, are able to regulate the deubiquitination process of BRI1 and BES1/BZR1, thereby affecting their stability and functionality [38,39]. KIB1 is an F-box protein that can mediate the degradation of BIN2, thereby relieving the inhibitory effect of BIN2 on BR signaling [40]. There are also studies indicating that RACK1, as a scaffold protein, can interact with BZR1 and enhance its nuclear localization by competitively reducing the interaction between BZR1 and 14-3-3 proteins [41,42]. A recent study has shown that atp-binding cassette b19 (ABCB19) functions as a brassinosteroid exporter and plays an important role in BR transport [29].These studies reveal the crucial role of these proteins in BR signaling, aiding in understanding the complex regulation of the signaling pathway (Figure 2).

BRs are perceived by the receptor kinase BRI1 and its co-receptor BAK1 on the plasma membrane. Upon binding, BRI1 undergoes autophosphorylation and forms a heterodimer with BAK1. The activated BRI1 phosphorylates its inhibitor BKI1, causing its dissociation from the plasma membrane and further activating BRI1 and BAK1. Subsequently, BRI1 phosphorylates downstream BR signaling kinases BSK1 and CDG1, initiating a phosphorylation cascade. After activation, BRI1 dephosphorylates and inhibits the GSK3-like kinase BIN2 via phosphatases such as BSU1, leading to the dephosphorylation of its substrates BZR1 and BES1. Dephosphorylated BZR1 and BES1 accumulate in the nucleus, bind to the promoters of BR-responsive genes, and regulate gene expression, thereby promoting plant growth and development. Additionally, the BR signaling pathway features negative feedback mechanisms, such as the degradation of BIN2 and the regulation of the phosphorylation status of BZR1/BES1, to maintain the homeostasis of the signaling pathway.

## 4. The Crosstalk Among BRs and Other Phytohormones in Brassicaceae Plants

### 4.1. The Synergistic Interaction Between BRs and Auxins

**Figure 3:**
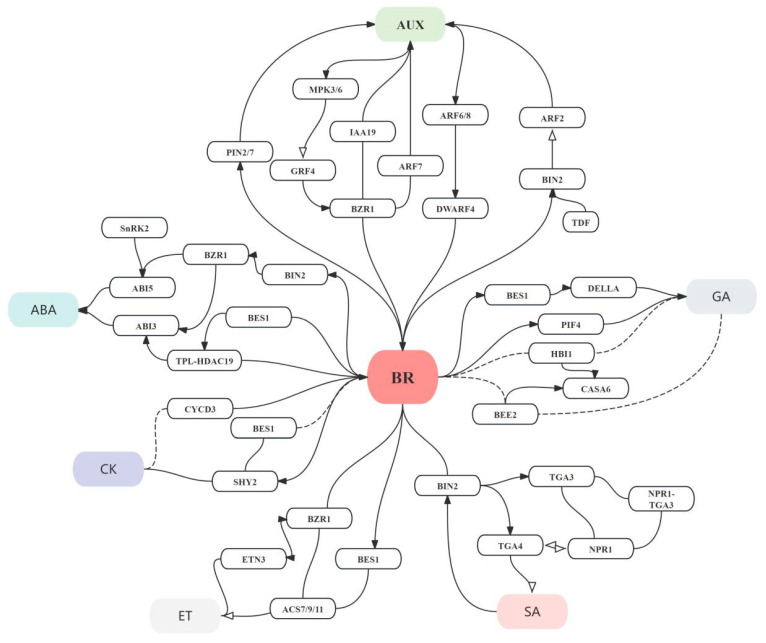
The gene regulation interaction of BR with AUX, ABA, GA, CK, ET, and SA in Brassicaceae plants.

Auxins and BRs are two major growth promoting phytohormones in plant development. Initial studies suggest that the signaling, biosynthesis, and transport of auxin and the signaling of BRs may be connected by upstream signals, such as calmodulin and phosphatidylinositol signaling [43]. Moreover, the actin cytoskeleton is an integration node for the BR signaling pathway and auxin responsiveness and the reconfiguration of actin filaments activates BR signaling, which in turn leads to enhanced auxin responsiveness [44]. Some studies have also found that BIN2 directly inactivates the repressor auxin response factor 2 (ARF2), thereby increasing the expression of auxin-induced genes and leading to a coordinated increase in transcription [45]. In arabidopsis, previous studies have shown that BR can regulate hypocotyl elongation through the auxin signaling-related components indole-3-acetic acid 19 (IAA19) and auxin response factor 7 (ARF7) [46]. auxin response factor 6 (ARF6) and auxin response factor 8 (*ARF8*) activate the transcription of *DWARF4*, which encodes a key BR biosynthetic enzyme. This indicates that auxin determines cell wall mechanics and directional cell growth through the biosynthesis of BRs, thereby producing leaves with variable roundness [47]. Recently, study has revealed that the molecular mechanism underlying auxin-mediated hypocotyl elongation involves the stability of the growth-regulating factor 4 (GRF4) protein regulated by mitogen-activated protein kinase 3/6 (MPK3/6) [48]. Tracheary element differentiation inhibitory factor (TDIF) initiated signaling through the TDR (TDIF receptor) directly acts on BIN2-mediated ARF phosphorylation, thereby regulating auxin signaling during lateral root development [49]. The BR signal controls the accumulation of PIN-LIKES in the endoplasmic reticulum, thereby increasing nuclear abundance and auxin signaling [50]. To illustrate, BRs regulate auxin transport and distribution in roots by modulating the endocytosis and intracellular distribution of the PIN2 protein, thereby controlling root growth and gravitropic responses in arabidopsis [51]. Additionally, recent studies have shown that the auxin transporter ABCB19 plays a significant role in BR output [52]. A recent publication indicates that exogenous BR treatment mainly promotes seedling establishment by up-regulating the expression of auxin-related genes during seed germination and seedling establishment stages in rapeseed [15]. Therefore, BRs and auxins have extensive synergistic effects in plant development, and they interact with each other through various molecular mechanisms at different developmental stages of plants (Figure 3).

### 4.2. The Interaction Between BR and ABA

The majority of research has shown that ABA exerts inhibitory effects on various aspects of plant growth and development, such as seed germination, cell elongation, and stomatal movement. As the second most important growth-promoting hormone after auxin, the antagonistic regulation of BRs with ABA has been well established [53]. For example, some studies have found that ABA induces the expression of ROS generating genes, while BR treatment can inhibit the production of ROS [54]. BIN2 can physically interact with ABI5 and phosphorylate it, thereby mediating the antagonism of BRs to ABA signaling in arabidopsis [55]. In addition, the interaction between BES1 and ABI5 can inhibit the binding of ABI5 to the promoter regions of downstream genes, thereby reducing their expression and ultimately promoting seed germination [56]. BES1 also can bind to the TPL-HDAC19 complex to inhibit the expression of *ABI3* in the ABA signaling pathway, thereby attenuating the ABA signal [57]. In the BR signaling pathway, the BIN2 kinase inhibits the activity of BZR1 through phosphorylation [58]. In the ABA signaling pathway, the activation of SnRK2 kinase can promote the expression of *aba insensitive 5* (*ABI5*) [59]. However, BES1/BZR1 can attenuate the ABA signal by inhibiting the expression of ABI3 and ABI5 in the ABA signaling pathway [60]. Although research has shown that antagonistic interactions exist between ABA and BR, some studies have also found evidence of synergistic interactions. The above studies indicate that ABA and BR not only have antagonistic mechanisms but also exhibit synergistic interactions during plant growth and development (Figure 3).

### 4.3. The Interaction Between BR and Gibberellins

BRs and gibberellins (GAs) promote cell elongation and expansion [61]. BRs facilitate cell elongation by regulating the relaxation of the cell wall and maintaining hormonal balance within the cell, while GAs achieve a similar effect by activating expansins in the cell wall [62]. For example, in Arabidopsis, BRs and GAs jointly regulate the elongation of the hypocotyl and stem, promoting rapid plant growth under low-light conditions [63]. Some studies have found that the dephosphorylated active BZR1 and BES1 can bind to DELLA (DELLA family proteins, the negative regulator of GA proteins), thereby enhancing GA-regulated cell elongation [64]. Furthermore, BR and GA treatment induces the expression of *HBI1* and *BEE2*. *HBI1* and *BEE2* interact with each other to synergistically activate the expression of *GASA6*, which in turn promotes endosperm rupture and seed germination in Arabidopsis [65]. Furthermore, BR, auxin, and GA regulate light-related hypocotyl elongation through the cooperative interaction of the BZR-ARF-PIF/DELLA (BAP/D) transcription factors/regulator in Arabidopsis [66]. Additionally, the elongation of the hypocotyl is promoted by GA, a process that relies on the participation of BR signaling, which regulates cell elongation through the phosphorylation of PIF4 [67]. Based on the above studies, BR and GA can promote the growth of Brassicaceae plants through various regulatory mechanisms (Figure 3).

### 4.4. The Interaction Between BR and Cytokinins

Cytokinins (CKs), as a class of phytohormones, play regulatory roles in various aspects of plant growth and development, including the growth of stems and roots, the development of chloroplasts, the filling of seeds, the aging process of plants, and the uptake of nutrients [68,69,70]. BR and cytokinins interact either synergistically or antagonistically to regulate plant growth and development [71]. EBR may promote cell division through CycD3 and can substitute for CK in the culture of arabidopsis callus and suspension cells [72]. The optimal root growth is regulated by the regulatory interactions among BRs, auxin, and CK through the genes *BES1*, *PINFORMED 7* (*PIN7*), and *short hypocotyl 2* (*SHY2*) [73]. Another important point is that the interaction between BR and CK promotes ovule initiation and increases seed number in arabidopsis [74] (Figure 3).

### 4.5. The Interactions Between BRs and Other Phytohormones

It is widely known that ethylene (ET) is a hormone that can accelerate plant senescence [75]. Moreover, there are also studies showing that it has a synergistic effect with BRs to promote plant growth and development. Some studies have shown that BRs and ET interdependently promote apical hook development and cell elongation through the direct interaction between BR-activated BZR1 and ET-activated EIN3 in arabidopsis [76]. The BR signaling transcription factors BES1 and BZR1 directly bind to the promoters of *ACS7*, *ACS9* and *ACS11*, repressing their expression, thereby reducing ethylene levels and promoting root growth in arabidopsis [77] (Figure 3).

By regulating the concentration of osmolytes, the levels of antioxidant enzymes, and the photosynthetic system, exogenously applied BR and jasmonic acid (JA) can help Brassica rapa alleviate drought stress [78]. Additionally, BRs are involved in the growth-defense tradeoff in arabidopsis by repressing the expression of JA-induced defensin and glucosinolate biosynthesis genes. Furthermore, BRs and JAs work together to balance the energy allocation between growth and defense responses [79,80]. JA-induced anthocyanin accumulation is affected by BR through its regulation of the “late” anthocyanin biosynthesis genes [81]. Currently, there are few reports on the molecular mechanisms of the interaction between BR and JA in Brassicaceae plants.

Salicylic acid (SA) is a class of phenolic hormones widely present in plants [82]. It not only regulates plant growth and development but also participates in plant immune and defense responses [83]. As a key negative regulator of BR signaling, BIN2 plays an important role in SA signaling [84]. Through BIN2, BR and SA helps plants achieve a balance between growth and immunity. On the one hand, BIN2 phosphorylates Ser-202 of tgacg motif-binding factor 4 (TGA4), inhibiting the redox-dependent interaction between TGA4 and pathogenesis-related genes (NPR1) and destabilizing TGA4, thereby negatively regulating SA-mediated plant immune responses [85]. On the other hand, salicylic acid activates the BIN2 kinase, which phosphorylates Ser33 of TGA3, enhancing TGA3’s DNA-binding ability and the formation of the nonexpressor of npr1-tga3 complex, leading to the activation of pathogenesis-related (PR) gene expression and enhanced disease resistance in plants [86] (Figure 3).

Solid black arrows indicate positive regulation, hollow black arrows indicate inhibitory regulation, lines without arrows indicate binding, and dashed lines indicate indirect mechanisms. The diagram is summarized from references [43,44,45,46,47,48,49,50,51,52,53,54,55,56,57,58,59,60,61,62,63,64,65,66,67,68,69,70,71,72,73,74,75,76,77,78,79,80,81,82,83].

## 5. BRs Shape the Environmental Adaptability in Brassicaceae Crops

### 5.1. BRs Regulate Temperature Stress Responses in Brassicaceae Crops

**Figure 4:**
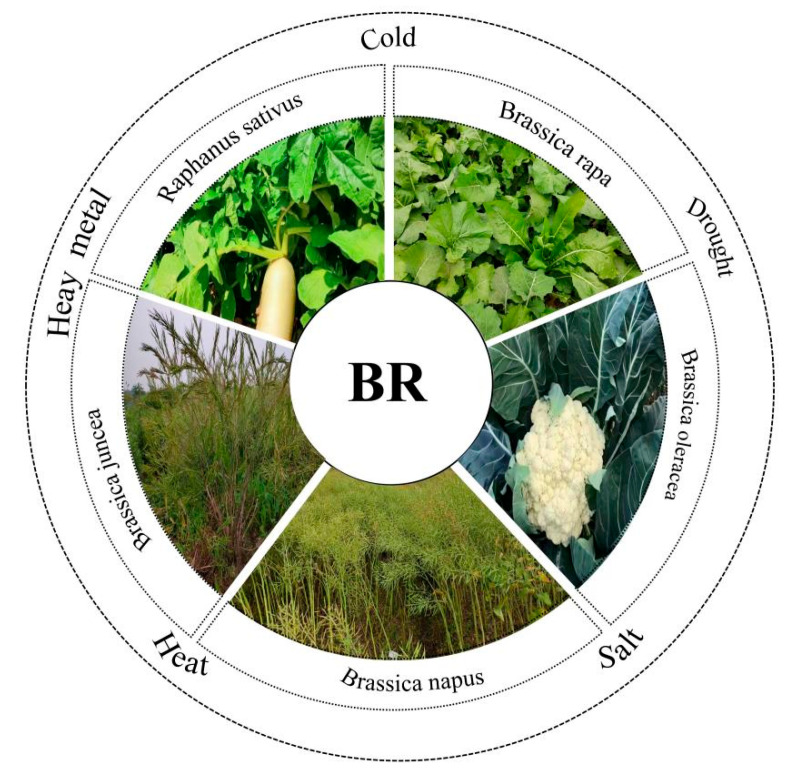
BRs shape the environmental adaptability in Brassicaceae crops.

Temperature affects the growth, development and geographical distribution of plants. Extreme temperatures (high and low) may cause irreversible damage to plant growth, development and productivity [87] (Figure 4). BRs play important roles not only in plant growth and development but also in plant environmental adaptation [88]. BRs control low-temperature stress responses. Studies have shown that the endogenous levels of BRs in plants increase under low-temperature stress [89]. Exogenous application of 24-epibrassinolide (EBR) significantly improves cold tolerance in plants [90]. In *Brassica rapa*, some studies showed that exogenous BR treatment can alleviate the damage caused by low temperature [91,92]. In *Brassica napus*, cold acclimation and deacclimation change brassinosteroid homeostasis [93,94]. Additionally, BRs enhance cold tolerance by regulating gene expression and activating the ICE-CBF-COR signaling pathway as well as non-CBF-dependent pathways [95]. In arabidopsis, EBR alleviates the inhibition of photosynthesis under low-temperature stress by regulating the expression of photosynthesis-related genes and enhancing the activity of antioxidant enzymes [96,97]. Low-temperature stress can lead to a decrease in photosynthetic efficiency. However, BRs can mitigate this effect through multiple mechanisms [98]. For example, treatment with EBR can increase the maximum quantum efficiency of photosystem II (PSII, Fv/Fm) in plants and reduce the decline in photosynthesis under low-temperature stress [99]. In addition, BRs enhance the photoprotective capacity of plants by activating the cyclic electron transfer dependent on PGR5 and antioxidant pathways [100]. To summarize, it is suggested that BRs play a significant role in ameliorating the adverse effects of low-temperature stress on plant growth and development.

BRs can not only alleviate the adverse effects of low temperature on plant growth and development, but also play an important role in plant responses to high temperature stress [101] (Figure 4). In *Brassica napus*, exogenous BR treatment of seedlings can significantly enhance their basal thermotolerance and lead to higher accumulation of four major classes of heat shock proteins (HSPs) [102]. In some case, BR augments thermotolerance in plants, but it is not necessary for hsp expression during HS [103]. Previous studies have already shown that EBR can enhance the thermotolerance of rapeseed (*Brassica napus*), and subsequent research has further demonstrated this ability [104,105]. Overexpression of the brassinosteroid biosynthetic gene *DWF4* in Brassica napus simultaneously increases seed yield, stress tolerance dehydration and heat stress, and enhanced resistance to necrotrophic fungal pathogens Leptosphaeria maculans and Sclerotinia sclerotiorum [106]. Seed priming with brassinolides improves growth and reinforces antioxidative defenses under normal and heat stress conditions in seedlings of *Brassica juncea* [43]. In summary, the above studies have demonstrated that BRs play an important role in alleviating the impact of temperature stress in Brassicaceae crops.

### 5.2. BRs Regulate Drought Stress Responses in Brassicaceae Crops

BRs are also involved in plants’ response to drought stress [107] (Figure 4). Drought tolerance in Brassicaceae is mostly determined by the increased endogenous levels of IAA, CKs, ABA and SA and the decreased levels of active BRs [75]. Exogenous BR and jasmonic acid improve drought tolerance in *Brassica rapa* genotypes by modulating osmolytes, antioxidants and photosynthetic system, and the application of exogenous BRs significantly alleviated drought stress in rapeseed by enhancing photosynthetic rate, photosynthetic pigments, stomatal conductance, transpiration rate, and antioxidant defenses [108]. Specificity in the root domain, accumulation of Phytoglobin1 and nitric oxide (NO) determines meristematic viability in water-stressed Brassica napus roots [109]. Overexpression of *brz insensitive long hypocotyl 9* (*BIL9*) enhances drought stress resistance through BR signaling [110]. In *Brassica rapa*, three BrBZRs gave co-responsive expression against cold, salt, and drought treatment, suggesting their multiple functions related to stress resistance [111].

### 5.3. BRs Regulate Salt Stress Responses in Brassicaceae Crops

Salt stress is one of the most severe abiotic stresses affecting plants, potentially reducing crop yields and impairing plant growth and development [112] (Figure 4). Many results indicate that BRs play a significant role in alleviating salt stress in plants, primarily by enhancing photosynthesis and reducing ROS [113]. For example, the application of exogenous BRs alleviated salt stress and maintained photosynthetic capacity, while also eliminating the production of ROS induced by salt stress in apple seedlings [114]. Additionally, exogenous BRs significantly enhance plant germination rates, plant height, root length, and biomass under salt stress conditions. Salinity induced contrasting changes in levels of the growth promoting hormones BRs in Kale (*Brassica oleracea*), Chinese cabbage and white cabbage [115]. These effects are achieved through the promotion of photosynthesis, increased activity of antioxidant enzymes, and the accumulation of osmo-protectants and antioxidants. BRs also reduce the content of harmful substances and sodium ions (Na^+^), thereby mitigating cellular damage and improving plant salt tolerance [116]. Studies have found that xyloglucan endotransglucosylase 19/23 (XTH19/23) are involved in lateral root development through the BES1-dependent pathway and contribute to the adaptation of lateral roots to salt stress in arabidopsis [117]. The BR biosynthesis gene *MdBR6OX2* positively regulates the salt tolerance of arabidopsis [118]. A certain high concentration of EBR can enhance the salt tolerance of rapeseed [119]. 28-EBR treatment improves the ability of mustard (*Brassica juncea*) to withstand the stress caused by the combined effects of temperature and salt stress by strictly regulating the accumulation of ROS [120]. These studies demonstrate that BRs can enhance the survival and productivity of Brassicaceae crops under salt stress conditions through a variety of physiological and molecular mechanisms.

### 5.4. BRs Respond to Metal Stresses in Brassicaceae Crops

BRs also play an important role in improving plant responses to metal stress. The harmful effects on plants can be mitigated by seed soaking and foliar application of BRs [121] (Figure 4). Calcium (Ca) deficiency triggered tip-burn through BR pathway in Chinese Cabbage (*Brassica rapa* L. ssp. Pekinensis) [122]. Exogenous BRs alleviate calcium deficiency induced tip-burn by regulating calcium transport in *Brassica rapa* [123]. Under lead (Pb) stress, exogenous BRs can increase the germination rate of seeds of *Brassica campestris* L. [111]. Consistently, exogenous BRs increase lead stress tolerance in seed germination and seedling growth of *Brassica juncea* [43]. The combined treatment of exogenous BRs and salicylic acid can reduce Pb uptake and increase the tolerance index to heavy metals, thereby alleviating the negative effects of Pb in *Brassica juncea* [124]. In addition, treatment with EBL increases the activities of antioxidant enzymes such as catalase and peroxidase, which help to counteract the toxic effects of Pb in *Brassica juncea* [43]. Epibrassinolide induces changes in indole-3-acetic acid, ABA and polyamine concentrations and enhances antioxidant potential of radish seedlings under copper (Cu) stress [125]. Co-application of EBR and spermidine is an effective approach for copper detoxification and the maintenance of homeostasis in *Raphanus sativus* [126]. The application of EBL and selenium (Se) can regulate the distribution of copper, enhance photosynthetic characteristics and increase the accumulation of various antioxidant enzymes and proline involved in photosynthesis in *Brassica juncea*, thereby achieving copper detoxification [105]. Chromium (Cr) stress mitigation by polyamine-brassinosteroid application involves phytohormonal and physiological strategies in *Raphanus sativus* L. [127]. The effect of 28-homobrassinolide on the antioxidant defense system in radish under chromium toxicity has been noted [128]. Foliar application of BRs alleviates adverse effects of zinc (Zn) toxicity in radish [129]. Taken together, exogenous BRs can mitigate the damage caused by metal stress in plants, primarily by reducing reactive oxygen species (ROS) and enhancing photosynthetic capacity.

BRs regulate cold, heat, salt, drought and heavy metal responses in Brassicaceae crops.

## 6. Conclusions

BRs regulate growth, development, and environmental adaptation in Brassicaceae crops, such as *Brassica rapa*, *Brassica oleracea* and *Brassica napus,* but this remains to be further explored. How BRs interact with other hormones at the cellular and tissue levels to regulate the growth, development, and environmental adaptation of *Brassica napus* and other Brassicaceae plants is still unclear. Therefore, further identification and elucidation of new BR regulatory components and a further understanding of hormone crosstalk and the molecular mechanisms of environmental adaptation are crucial for advancing the genetic improvement of Brassicaceae crops. For instance, detailed elucidation of the molecular mechanisms of BR and auxin interactions under low-temperature conditions could have significant practical value for expanding the cultivation of Brassica napus in cold regions.

## 7. Perspectives

Phytohormones influence the growth, development, and environmental adaptation of plants through various regulatory networks during their growth process [130,131,132]. In recent years, research has greatly deepened our understanding of the role of BRs and hormone interactions in shaping the agronomic traits of Brassicaceae plants. However, this understanding is still limited. Recently, the BR signaling pathway has gradually been elucidated, especially in arabidopsis [133]. As a key plant hormone, BRs play important roles in many growth and development stages and physiological activities of plants [134]. They are widely distributed in various parts of plants, such as pollen, seeds, stems, and leaves, and are synthesized through complex metabolic pathways, thereby regulating plant growth, development, and adaptability to the environment [135,136,137]. Recent studies have reported that the auxin transporter ABCB19 in arabidopsis can export BRs to the apoplast, further elucidating the close connections between phytohormones [29,45]. Additionally, the interplay between BRs and other hormones in plant growth, development, and environmental adaptation has been extensively documented, such as the promotion of hypocotyl elongation, seed germination, and enhancing stress resistance. However, these reports are still insufficient, and the molecular mechanisms by which BRs coordinate with other hormones to regulate these processes require further investigation.

## Data Availability

Not applicable.
